# Prevalence and Risk Factors for Augmented Renal Clearance in Neurocritical Ill Patients

**DOI:** 10.1002/jcla.70047

**Published:** 2025-05-20

**Authors:** Xiangru Ye, Qiang Yuan, Zhuoying Du, Haijun Yao, Lei Yang, Biwu Wu, Gang Wu, Weilin Shi, Yufeng Jin, Zhiping Liu, Caihua Xi, Jin Hu

**Affiliations:** ^1^ Department of Neurosurgery & Neurocritical Care, Huashan Hospital Fudan University Shanghai China; ^2^ Research Unit of New Technologies of Micro‐Endoscopy Combination in Skull Base Surgery (2018RU00) Chinese Academy of Medical Sciences Beijing China; ^3^ Nursing Department, Huashan Hospital Fudan University Shanghai China

**Keywords:** augmented renal clearance, critical care, neurosurgery, prevalence, renal elimination, risk factors

## Abstract

**Background:**

Augmented renal clearance (ARC) refers to a phenomenon in critically ill patients characterized by increased creatinine clearance. Neurological patients seem to be at higher risk compared with other groups. The epidemiology study of ARC reported in critically ill neurological patients varies substantially with the definitions used and the population evaluated.

**Objective:**

We aimed to describe the prevalence of ARC and to explore risk factors in critically ill neurological patients.

**Methods:**

A retrospective observational study was conducted in a university‐affiliated neurocritical care unit (NCCU). Study participants had a serum creatinine concentration (Scr) < 120 μmol/L. Kidney function was assessed by the 24‐h creatinine clearance (CL_cr_); ARC was defined as CLcr ≥ 120 mL/min/1.73m^2^ in women and ≥ 130 mL/min/1.73m^2^ in men. The prevalence and clinical characteristics of ARC were evaluated. Multivariate logistic regression analysis was used to assess variables associated with ARC occurrence.

**Results:**

Of the 137 patients, 56.2% were male, and the mean age was 50.2 (17.4) years. ARC was present in 55.5% of the NCCU patients, ranging from 50% in intracranial infection to 75% in patients with spinal lesions. ARC patients have a mean CL_cr_159.3 (IQR:139.6–185.2) ml/min/1.73m^2^. Age was the only factor independently associated with ARC (OR 0.996, 95% CI: 0.934–0.999, *p* = 0.043) in multivariable logistic analysis. Scr (Pearson correlation = −0.477) and cystatin C (Pearson correlation = −0.336) were found to have a negative correlation with ARC with statistically significant effects.

**Conclusion:**

ARC is prevalent in critically neurological patients. Age is likely to significantly influence renal clearance in this population, especially as patients with low Scr and cystatin C levels should be given more attention.

## Graphical abstract

Augmented renal clearance is prevalent in critically neurological patients. Age is likely to significantly influence renal clearance in this population, especially patients with low Scr and cystatin C levels should be given more attention.

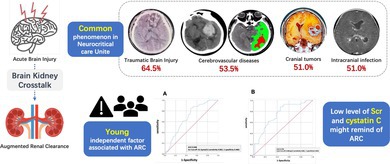

## 1 Background

Critically ill patients represent a unique population, distinct from those seen in outpatient or general ward settings. As acute kidney injury (AKI) is a common complication in these individuals, with an incidence ranging from 35% to 38% [1], clinicians are therefore vigilant in adjusting the doses of renally eliminated drugs in patients with varying degrees of renal impairment, using mathematical formulae to estimate the glomerular filtration rate (GFR) [2] and minimize the risk of potential drug toxicities. In contrast, less attention has been paid to the phenomenon of augmented renal clearance (ARC) in intensive care units (ICU), despite its widespread occurrence. This often overlooked phenomenon has earned the description of “the elephant” in the ICU [3].

ARC is a recently recognized condition in critically ill patients characterized by increased creatinine clearance (CL_Cr_) and enhanced elimination of renally cleared medications. Patients with sepsis, trauma, and burns are consistently identified as being at risk for ARC [4, 5, 6, 7], with mean CL_Cr_ values ranging from 170 mL/min/1.73m^2^ to more than 300 mL/min/1.73m^2^ [8]. The incidence in these populations ranges from 18% to 80%, with particularly high prevalence in patients with traumatic brain injury (TBI, 85%) and subarachnoid hemorrhage (SAH, 100%), although the number of cases in the cohorts is limited [3, 7, 8, 9, 10].

The significance of ARC is increasingly recognized in neurocritical care [11]. Much of the existing literature has focused on the impact of ARC on the pharmacokinetics of antibiotics, including aminoglycosides, vancomycin, carbapenems, piperacillin, and quinolones [2, 10, 12]. Enhanced drug clearance in the setting of ARC leads to a shorter drug half‐life, lower peak drug concentration (C_max_), and reduced area under the concentration curve (AUC), all of which can directly affect a drug's pharmacodynamic effects, potentially resulting in therapy failure. Beyond antibiotics, ARC can also influence the pharmacokinetics of other renally cleared medications, such as low‐molecular‐weight heparins and antiseizure medications [13, 14]. For instance, levetiracetam has been shown to undergo faster systemic clearance, and a shorter terminal elimination half‐life was observed in neurocritical care patients [15, 16]. In the context of ARC, standard dosing regimens may be inadequate, leading to adverse clinical outcomes such as treatment failure, which can manifest as complications like venous thrombosis or inadequate treatment of infections. This highlights the importance of considering ARC when determining treatment for infections. However, a survey of ICU physicians in England revealed that only 15% of respondents would modify the dosing regimen of beta‐lactams and vancomycin in patients with ARC [10].

Despite the growing recognition of ARC, large‐scale studies on its prevalence and associated risk factors in critically neurological patients are still lacking. The objective of this study is to describe the prevalence of ARC in the neurocritical care unit (NCCU) and evaluate the risk factors associated with its development.

## 2 Methods

### 2.1 Setting

This retrospective, observational cohort study was conducted in the Department of Neurosurgery & Neurocritical Care, Huashan Hospital, Fudan University between June 2019 and January 2020. The study protocol (KY2024‐1268) was approved by the institutional ethics committee of Huashan Hospital.

### 2.2 Study Population

Patients in our neurosurgical intensive care unit who met the following criteria were included in the study: (1) ≥ 16 years old; (2) had a timed urine collection assessment for CL_Cr_; (3) a plasma creatinine < 120 μmol/L, and no history of prior renal replacement therapy or chronic kidney disease (CKD).

### 2.3 Data Collection and Analysis

Each patient's medical record was reviewed to collect demographic, clinical, and outcome data, including age, gender, height, weight, admission diagnosis, ICU and hospital LOS, and mortality. The observed range of CL_cr_ in each diagnostic subgroup was analyzed. Additional data collected included admission days, Glasgow Coma scale (GCS) score, maximum body temperature, concomitant medications (dopamine, noradrenaline glucocorticoid, glycerin fructose, mannitol, diuretic), APACHE II (Acute Physiology and Chronic Health Evaluation II) and SOFA (Sequential Organ Failure Assessment) score, and laboratory tests serum creatinine concentration (Scr), cystatin C, leukocyte count, and proBNP (pro‐B‐type natriuretic peptide).

Given that derived estimates of GFR are inaccurate in the setting of ARC [17], a minimum collection period of at least 8 h is recommended for the determination of CL_cr_ [4]. A 24‐h measured CL_cr_ was obtained in our study using the following methods. Urine was collected via indwelling catheter, following which urinary volume and creatinine concentration were determined by laboratory analysis. Concurrent plasma creatinine was obtained at a point mid‐way through the urinary collection, following which CL_cr_ was calculated using the formula listed below [18].

$$\mathrm{BSA}=0.07184\times {\left(\mathrm{Ht}\right)}^{0.725}\times {\left(\mathrm{Wt}\right)}^{0.425}$$

$${\mathrm{CL}}_{\mathrm{Cr}}=\left(U_{\mathrm{Cr}}\times \text{Uvol}/P_{\mathrm{Cr}}\times 1440\right)\times 1.73/\mathrm{BSA}$$

*BSA* = body surface area (m^2^); *Ht* = height (cm), *Wt* = weight (kg), *CL*_*Cr*_ = 24‐h creatinine clearance (ml/min/1.73m^2^), *U*_*Cr*_ = urinary creatinine concentration (μmol/L), *U*_*vol*_ = urinary volume (ml), *P*_*Cr*_ = plasma creatinine concentration (μmol/L). ARC was defined as a 24‐h *CL*_*Cr*_ ≥ 130 mL/min/1.73m^2^ in men and ≥ 120 mL/min/1.73m^2^ in women.

### 2.4 Statistical Analysis

Continuous data are presented as the mean (SD) or median (interquartile range (IQR)), depending on the distribution. Categorical data are presented as counts (%). Comparisons of continuous data utilized a paired Student's t‐test. A logistic regression model was developed to assess the risk factors associated with ARC, both in univariable and multivariate analyses (backward conditional logistic regression model). Since Scr, cystatin, and CL_Cr_ have different units and show wide variability, we performed logarithmic transformations and analyzed correlations using Pearson correlation tests. Since the number of samples with missing data is small, we used listwise deletion to handle the missing data during statistical analysis. A two‐sided *p* < 0.05 was considered statistically significant. All analyses were conducted using SPSS version 26 (IBM Corporation, Armonk, New York).

## 3 Results

A total of 137 patients were included in the study, with all participants completing the 24‐h urinary CL_cr_ assessment. Demographic, admission diagnosis, concomitant disease, GCS, and laboratory tests are presented in Table 1. As shown, approximately 56.2% (77/137) of the cohort was male, with a mean age of 50.2 ± 17.4 years; only 25.5% of patients were ≥ 65 years old. More than one‐third (44.1%) of patients required invasive mechanical ventilation and 7.6% needed vasopressor support. The median length of hospital stays, ICU stay, and days after the most recent surgery were 9.0 (4.0, 15.0) days, 4.0 (3.0, 6.0) days, and 3.0 (5.0, 8.0) days, respectively. Regarding admission diagnosis, spinal lesion, intracranial infection, TBI, cerebrovascular diseases, and cranial tumors counted for 2.9%, 5.8%, 22.6%, 31.4%, and 37.2%, respectively. Of the patients, 98 (71.5%) had undergone prior surgery, and 78 (56.9%) were with GCS ≤ 8.

**TABLE 1:** Demographic characteristics of patients with and without augmented renal clearance (CL_cr_ ≥ 120 mL/min/1.73m^2^ (female), ≥ 130 mL/min/1.73m^2^ (male)).

	Total	ARC	Without ARC	*p*	95% CI
**Patients**	**137**	**76 (55.5%)**	**61 (45.5%)**		
Male, *n* (%)	77 (56.2%)	38 (50%)	39 (63.9%)	0.102	
Age (yr)	50.2 ± 17.4	44.8 ± 15.6	57.0 ± 17.4	**< 0.001**	6.57–17.73
Patients with age ≥ 65 yr., *n* (%)	35 (25.5%)	9 (11.8%)	26 (42.6%)	**< 0.001**	
Body surface area (m^2^)	1.7 ± 0.2	1.7 ± 0.2	1.7 ± 0.2	0.661	−0.05 to 0.78
Admission days in hospital (d)	9.0 (4.0, 15.0)	10.5 (6.5, 17.0)	12.5 (9.3, 19.8)	9.0 (4.0, 15.0)	−0.49 to 5.44
Admission days in ICU (d)	4.0 (3.0, 6.0)	5.0 (3.8, 6.0)	5.0 (3.0, 7.2)	4.0 (3.0, 6.0)	−0.49 to 5.44
Days to the latest operation (d)	3.0 (5.0, 8.0)	5.0 (4.0, 11.0)	6.0 (3.0, 11.0)	3.0 (5.0, 8.0)	1.56–3.26
Receive operation within 2‐week, *n* (%)	98(71.5%)	54(71.1%)	44(72.1%)	0.899	
**ICU admission group diagnosis**					
Intracranial infection, *n* (%)	8 (5.8%)	4 (5.3%)	4 (6.6%)	0.748	
TBI, *n* (%)	31 (22.6%)	20 (26.3%)	11 (18%)	0.250	
Cranial tumors, *n* (%)	51 (37.2%)	26 (34.2%)	25 (41%)	0.415	
Cerebrovascular disease, *n* (%)	42 (30.7%)	23 (30.3%)	19 (31.1%)	0.911	
Spinal lesions^a^, *n* (%)	4 (2.9%)	3 (3.9%)	1 (1.6%)	0.425	
Others, *n* (%)	1 (0.7%)				
**Comorbidity**					
Hypertension, *n* (%)	38 (27.7%)	14 (18.4%)	24 (39.3%)	**0.008**	
Diabetes/IGT, *n* (%)	18 (13.1%)	9 (11.8%)	9 (14.8%)	0.616	
CHD, *n* (%)	3 (2.2%)	0 (0%)	3 (4.9%)	0.051	
SOFA score	4 (3,6)	4 (2.5,5)	5 (3,7)	**0.006**	
APACHE II score	7 (5,9)	6 (4,8)	9 (6,10)	**< 0.001**	
**Concomitant medications**					
Glycerin fructose, *n* (%)	16 (11.8%)	6 (7.9%)	10 (16.7%)	0.115	
Mannitol, *n* (%)	78 (57.4%)	46 (60.5%)	32 (53.3%)	0.400	
Diuretic, *n* (%)	16 (11.8%)	6 (7.9%)	10 (16.7%)	0.115	
Torasemide, *n* (%)	4 (2.9%)	1 (1.3%)	3 (5.0%)	0.207	
Furosemide, *n* (%)	9 (6.6%)	4 (5.3%)	5 (8.3)	0.475	
Glucocorticoid, *n* (%)	43 (31.6%)	24 (31.6%)	19 (31.7%)	0.991	
Mineral corticoid, *n* (%)	23 (16.9%)	13 (17.1%)	10 (16.7%)	0.946	
Vasoactive drug, *n* (%)	9 (7.6%)	4 (6.3%)	5 (9.1%)	0.576	
**Clinical characteristics**					
GCS	8.0 ± 3.6	8.5 ± 4.0	7.4 ± 2.9	0.073	
GCS ≤ 8, *n* (%)	78 (56.9%)	40 (52.6%)	38 (62.3%)	0.256	−2.26 to 0.14
T_max_ (°C)	37.7 ± 0.7	37.7 ± 0.7	37.7 ± 0.7	0.953	−0.25 to 0.26
Fever (T ≥ 37.5°C), *n* (%)	63 (57.8%)	32 (54.2%)	31 (62.0%)	0.414	
IMV, *n* (%)	52 (44.1%)	26 (41.3%)	26 (47.3%)	0.512	
24‐h fluid balance (ml)	631.6 ± 659.4	567.3 ± 688.4	710.8 ± 618.2	0.212	−82.62 to 369.58
**Laboratory test**					
Measured 24 h‐CL_cr_ (ml/min/1.73m^2^)	130.7 (104.4, 165.9)	159.3 (139.6, 185.2)	101.6 (82.2, 114.0)	**< 0.001**	−88.19 to −64.99
ProBNP (pg/ml)	332.0 (162.7, 630.7)	268.5 (134.1, 520.1)	538.3 (322.7, 860.9)	0.129	−88.74 to 689.82
Scr (μmol/L)	55.1 ± 18.3	50.0 ± 14.1	61.5 ± 20.9	**< 0.001**	5.28–17.7
CsyC (mg/L)	0.7 (0.5, 1.0)	0.6 (0.5, 0.8)	0.9 (0.8, 1.1)	**0.001**	0.11–0.39
CRP (mg/L)	36.7 (17.2, 93.2)	34.9 (11.3, 77.8)	43.7 (17.9, 103.0)	0.142	−7.06 to 47.4
Leukocyte count (×10^9^/L)	10.3 (8.4, 13.8)	8.5 (6.8, 11.0)	11.4 (8.9, 14.7)	0.695	−1.27 to 1.91
hct (%)	31.8 (28.2, 35.5)	32.0 (28.4, 34.3)	31.6 (28.0, 35.1)	0.878	−1.93 to 1.65
Serum sodium (mmol/L)	142.0 (138.0, 144.0)	141(136, 143)	142 (139, 145.3)	0.143	−0.48 to 3.31
**Outcomes**					
Mortality (%)	**1 (0.73%)**	0	1	0.266	
ICU LOS (d)	11.0 (6.0, 17.0)	11.0 (5.8, 16.3)	16.5 (9.0, 25.3)	0.147	−0.94 to 6.19
Hospital LOS (d)	23.0 (15.8, 32.0)	29.5 (20.8, 35.0)	30.5 (22.5, 46.0)	0.348	−2.63 to 7.41

*Note:* Continuous variables that follow a normal distribution are expressed using the mean ± SD, while continuous variables that do not follow a normal distribution are expressed using the median (interquartile range, IQR). Significance of bold values indicate statistical significance (*p* < 0.05) or highlights primary outcomes.

Abbreviations: APACHE II, Acute Physiology and Chronic Health Evaluation; CHD, coronary heart disease; GCS, Glasgow Coma Scale; hct, hematocrit; IGT, impaired glucose tolerance; IMV, invasive mechanical ventilation; IQR, interquartile range; LOS, length of stay; ProBNP, Pro B‐type natriuretic peptide; SOFA, Sequential Organ Failure Assessment; Tmax, the maximum of body temperature in the 24‐h CL_cr_ sampling day.

^a^ Spinal lesions refer to any abnormality or damage in the spine, including conditions like tumors, fractures, or vascular malformation.

**FIGURE 1:**
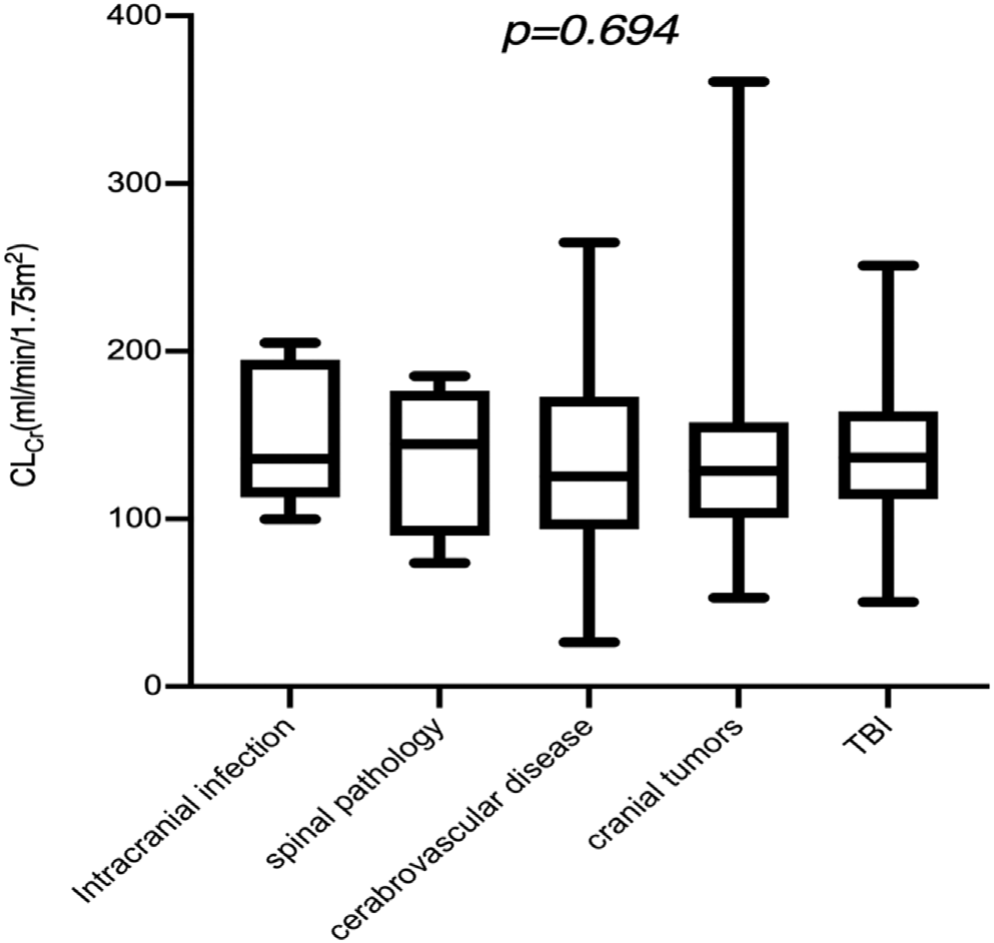
Different populations exhibiting ARC.

The prevalence of ARC in our NCCU was 55.5% (76/137), with the highest in patients with spinal lesions (3/4), followed by TBI (64.5%), cerebrovascular diseases (53.5%), cranial tumors (51.0%) and intracranial infections (50%). No significant difference in ARC prevalence was observed across different ICU admission diagnoses (Figure 1). A comparison of patients with and without ARC is shown in Table 1, highlighting the distinct characteristics of both groups. Compared with the non‐ARC group, APACHE II (4(2.5, 5) vs. 5 [3, 7], *p* = 0.006) and SOFA (6 [4, 8] vs. 9 [6, 10], *p* < 0.001) scores were lower in ARC patients; those with ARC had higher GCS (8.5 ± 4.0 vs. 7.4 ± 2.9) but a lower prevalence of concomitant medications like hypertension (18.4% vs. 39.3%), diabetes/impaired glucose tolerance (11.8% vs. 14.8%) or coronary heart diseases (0% vs. 4.9%), although none of these differences were statistically significant. Multivariate analysis identified younger age as the only factor significantly associated with ARC (OR 0.966, 95% CI: 0.934–0.999, *p* = 0.034, Table 2).

**TABLE 2:** Variable associated with ARC (Logistic regression, multivariate analysis).

Factors	OR	95% CI	p
Age (yr)	0.966	0.934–0.999	**0.043**
APACHE II score	0.947	0.764–1.173	0.616
SOFA score	0.865	0.700–1.069	0.179
Hypertension	0.655	0.267–1.607	0.356

*Note:* Significance of bold values indicate statistical significance (*p* < 0.05) or highlights primary outcomes.

Abbreviation: 95% CI, 95% confidence index.

**FIGURE 2:**
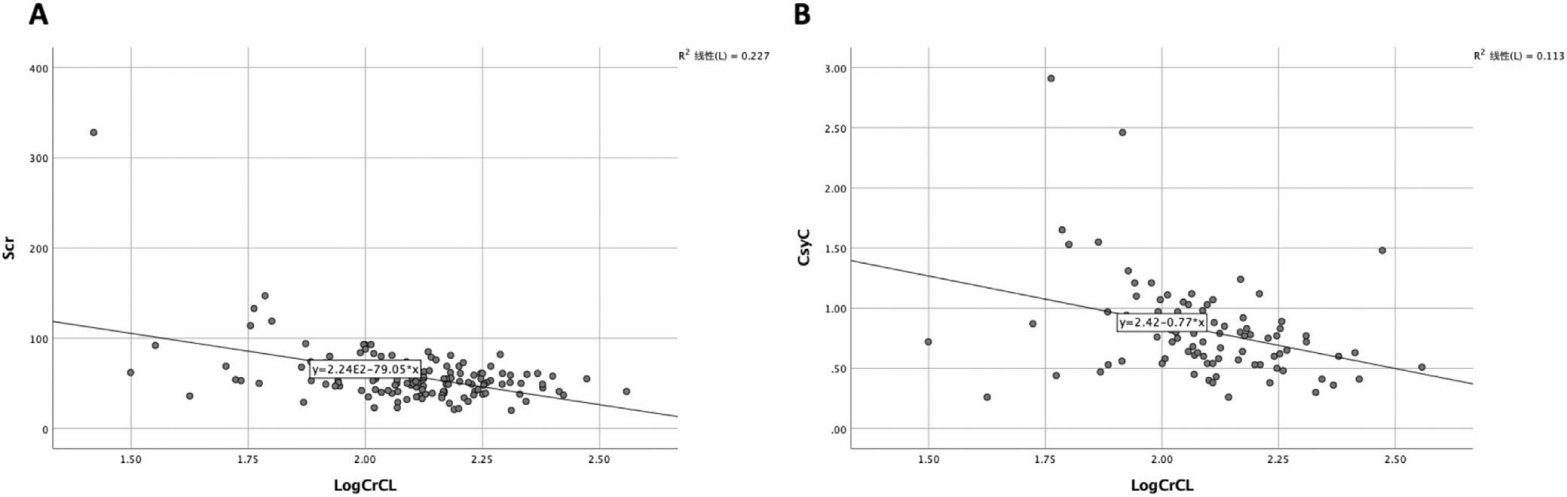
Correlation between Log (CL_Cr_ (ml/min/1.73m^2^)) and Cys C (mg/L), Scr (μmol/L). Scatter graphs of Log CL_cr_, and cystatin C (CysC, **A**), serum creatinine concentration (Scr, **B**) in neurocritical care patients. The Pearson correlation coefficient for Scr *r* = −0.477(*p* = 0.000), CysC *r* = −0.336(*p* = 0.02).

**FIGURE 3:**
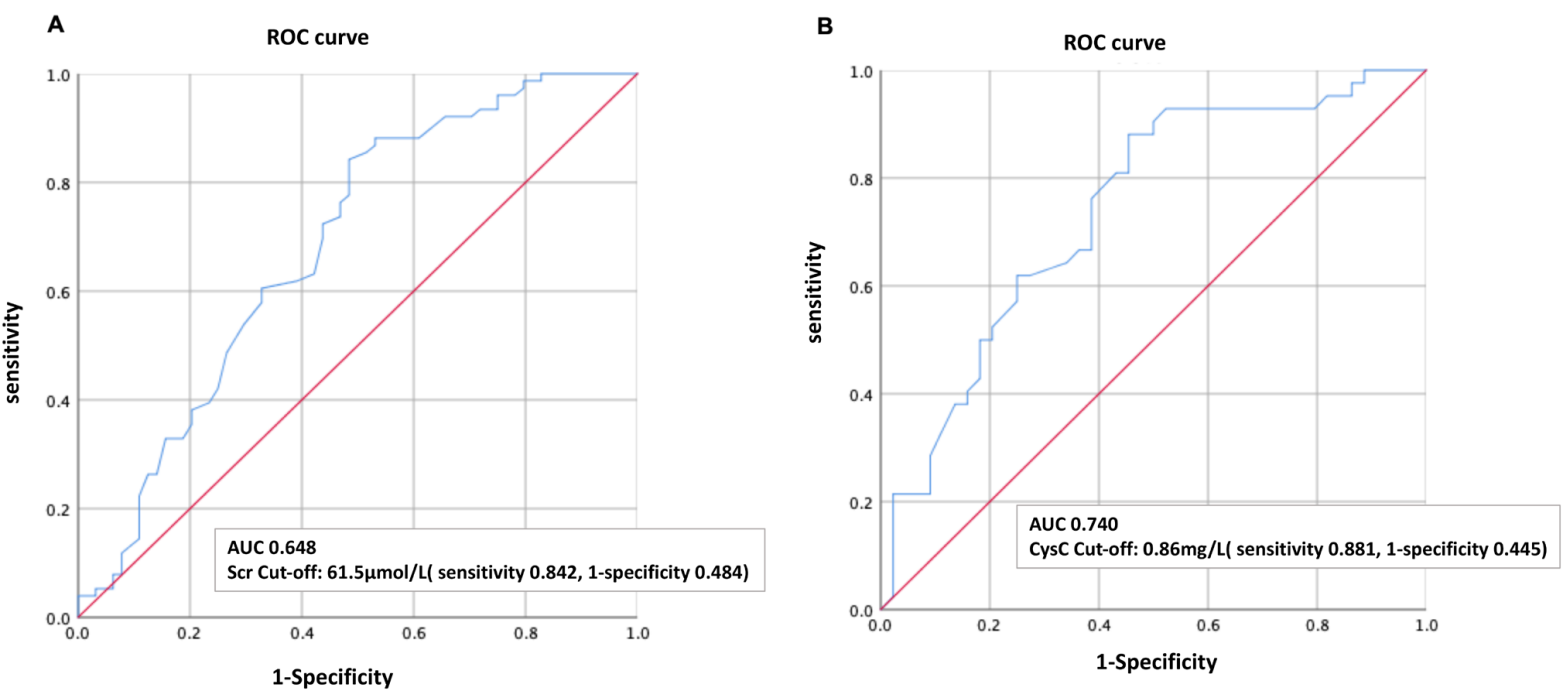
Receiver‐operator characteristic curves for detection of ARC (A) serum creatinine; (B) cystatin C. AUC: Area under the curve.

Univariate regression analyses of the mean Scr (50.0 ± 14.1 μmol/L vs. 61.5 ± 20.9 μmol/L, *p* = 0.000, 95% CI: 5.28–17.7) and cystatin C (0.6(0.5, 0.8) mmol/L vs. 0.9 (0.8, 1.1) mmol/L, *p* = 0.001, 95% CI: 0.11–0.39) revealed a strong negative and statistically significant association with ARC. These findings indicate that Scr and cystatin C—two commonly used biomarkers for assessing GFR—also showed a negative correlation with CL_cr_. However, their efficiency in identifying ARC was relatively low, with correlation coefficients of *r* = −0.477 and *r* = −0.336, respectively (Figure 2). Receiver‐operating characteristic (ROC) analysis showed that both biomarkers had over 80% sensitivity but relatively low specificity for identifying ARC (Figure 3).

## 4 Discussion

ARC is increasingly recognized in neurocritical care practice. To date, studies on ARC have primarily focused on hemorrhagic stroke [19, 20, 21], TBI [11, 22]. In our retrospective observational study, we analyzed a cohort of NCCU patients, including diagnostic subgroups such as TBI, cerebrovascular diseases (including both hemorrhagic and ischemic stroke), unruptured intracranial aneurysms, moyamoya disease, intracranial infection, cranial tumors, and spinal lesion, which represented 5.8%, 37.2% and 2.9% of the cohort, respectively. Our findings align with previous studies exploring the epidemiology of ARC in TBI and hemorrhagic stroke, confirming the high prevalence of ARC in critically neurological patients. A substantial proportion of NCCU patients (55.5%) developed ARC, with the highest prevalence in patients with spinal lesions (3/4), followed by TBI (64.5%), cerebrovascular diseases (53.5%), cranial tumors (51.0%) and intracranial infection (50%). In these patients, the 24‐h CL_Cr_ was 162.1 (147.3, 194.6) ml/min/1.73m^2^ in men and 155.3 (132.8, 180.7) ml/min/1.73m^2^ in women. The mechanisms underlying ARC in critically neurological patients remain incompletely defined. A plausible explanation could involve the neuro‐renal axis. Brain injury is associated with the release of inflammatory mediators, and current treatment protocols emphasize the importance of maintaining adequate cerebral perfusion with vasopressors, fluids, and hypertonic saline solutions [8, 13, 23, 24]. These interventions can influence renal perfusion and glomerular filtration. Additionally, the activation of “renal functional reserve” in younger patients may contribute to the development of ARC [25].

There is no consensus on the most accurate technique to quantify ARC at the bedside, or the “normal” reference values for any given population, which complicates the identification of ARC prevalence in ICU patients. The most widely accepted measure of renal function is the GFR or CL_Cr_. Many studies defined ARC as patients with CL_Cr_ > 130 mL/min/13m^2^, but other CL_Cr_ thresholds have been proposed, including CL_Cr_ > 120 mL/min/1.73m^2^, > 160 mL/min/1.73m^210^. Andrew A. Udy suggested defining ARC using GFR values approximately 10% above the upper normal limit, which translates to > 160 mL/min/1.73m^2^ in men and > 150 mL/min/1.73m^2^ in women [13]. Given the variability of normal GFR values based on age, gender, and body size, we adopted thresholds of > 130 mL/min/1.73m^2^ for men and > 120 mL/min/1.73m^2^ for women, which have also been used in other studies [8, 20].

Recognizing the risk factors for ARC allows clinicians to identify at‐risk patients. Various factors have been explored in critically ill settings, including trauma, illness severity as assessed by the APACHE II score, simplified acute physiology (SAPS II) and/or SOFA score. Some factors were initially considered risks for ARC in univariate analysis but were not subsequently confirmed after statistical adjustment. These include male sex, mechanical ventilation, high diastolic blood pressure, high cardiac index, vasopressor use (both high and low), low use of furosemide, high diuretic volumes, and a less positive fluid balance [7]. The most consistent risk factor associated with ARC is younger age. In our multivariate analysis, age was the only factor independently associated with ARC after adjusting for other factors available in our medical records. While patients with ARC had higher GCS, no statistically significant difference was found.

Identifying the optimal surrogate marker for ARC is a challenging task. ARC is clinically silent, and only accurate measures of urinary clearance (at least 8 h collection for CL_cr_) can reliably identify this patient group [4]. Mathematically, estimates of GFR, such as those based on C&G (Cystatin C with the Cockcroft and Gault formula), CKD‐EPI equation, MDRD‐4, or MDRD‐6 scores of glomerular filtration rate, have proven inadequate and inaccurate in detecting ARC [2, 17]. Currently, most ICUs use Scr as the standard measure of kidney function due to its high specificity and low cost, despite low sensitivity [26]. Cystatin C has been used for approximately 40 years as an endogenous marker of GFR and is preferred over Scr in some clinical settings [27]. However, it still lacks sufficient sensitivity in detecting ARC and is rarely used in clinical practice outside a few centers [8, 28, 29]. In our study, both Scr and Cys C levels showed negative correlations with CL_cr_, which could help alert clinicians to the presence of ARC.

Several limitations should be considered when interpreting this study. First, the retrospective nature of data means that the quality of the information depends on the accuracy of the recorded data. This was a single‐center study, so the findings may not be generalizable to other clinical settings. Second, 24‐h CL_cr_ was only measured once in our patients. As part of routine care, timed urine collections are conducted for NCCU patients at least once when the anticipated length of stay exceeds 48–72 h, in accordance with our institutional protocol during the study time. Since the onset and duration of ARC are not well defined, it likely varies among patients and depends on the underlying disease process and clinical interventions employed. Udy et al. found that 38% of patients had ARC on the first day of ICU admission [30]; and peak CL_cr_ values in in TBI were recorded after a mean of 4.7 days [13]. Fuster Liuch et al., observed that the highest prevalence of ARC on day 5 [31], While Brown et al. found peak CL_cr_ levels on the fourth day, with levels returning to baseline by day 7 [32]. Although many studies that ARC can persist for weeks, others document transient ARC lasting no more than 1 day [10]. Our data showed that the shortest day and longest durations for detecting ARC were 1 day and 24 days after ICU admission, and 1 day and 34 days after surgery, respectively. Further research is needed to explore the time course of ARC more comprehensively. Thirdly, power analysis and sample size calculation were not performed prior to the study, but there were no ethical constraints requiring a minimum number of patients for inclusion given the observational nature of the study.

## 5 Conclusion

In the present study, we observed a high prevalence of ARC (55.5%) in this cohort of critically ill neurological patients, with the prevalence especially high in a subgroup of patients with TBI, where it reached 64.5%. Younger age was the only factor that independently predicted ARC condition. Lower Scr and Cys C levels, despite being nonmodified, were correlated with ARC, can be easily recognized, and help to identify the risk of ARC.

## Ethics Statement

The study was approved by the institutional ethics committee of Huashan Hospital (approval number: KY2024‐1268).

## Conflicts of Interest

The authors declare no conflicts of interest.

## Data Availability

The data that support the findings of this study are available from the corresponding author upon reasonable request.
